# Examining the Schelling Model Simulation through an Estimation of Its Entropy

**DOI:** 10.3390/e20090623

**Published:** 2018-08-21

**Authors:** Alexander V. Mantzaris, John A. Marich, Tristin W. Halfman

**Affiliations:** Department of Statistics, University of Central Florida (UCF), TC2 4000 Central Florida Blvd, Orlando, FL 32816-2370, USA

**Keywords:** Schelling model, spatial analysis, entropy, 91-08, 62M30, 91D10

## Abstract

The Schelling model of segregation allows for a general description of residential movements in an environment modeled by a lattice. The key factor is that occupants change positions until they are surrounded by a designated minimum number of similarly labeled residents. An analogy to the Ising model has been made in previous research, primarily due the assumption of state changes being dependent upon the adjacent cell positions. This allows for concepts produced in statistical mechanics to be applied to the Schelling model. Here is presented a methodology to estimate the entropy of the model for different states of the simulation. A Monte Carlo estimate is obtained for the set of macrostates defined as the different aggregate homogeneity satisfaction values across all residents, which allows for the entropy value to be produced for each state. This produces a trace of the estimated entropy value for the states of the lattice configurations to be displayed with each iteration. The results show that the initial random placements of residents have larger entropy values than the final states of the simulation when the overall homogeneity of the residential locality is increased.

## 1. Introduction

Human actors are very complex objects to model and the factors of the environment which affect their behavioral state is a challenge to encapsulate in a mathematical framework. This difficulty is further increased when long term macroscopic changes are taken into account. Modeling residential patterns as they evolve is of great interest, as the housing market is a large part of the economy, and the spatial arrangements shape the interactions among community members. The Schelling model of segregation [1], presents a model of residential movement dynamics based upon a homophilic threshold of satisfaction (Appendix A) for the number of similarly labeled neighbors in a grid. The model can be described as a set of hypothetically labeled residents in a grid allotment which, during a simulation, proceeds to be given a random position allocation when there are not enough of their adjacent neighbors sharing the same label. A simulation continues to allocate random position reassignments until all residents have the required amount of identically labeled neighbors needed to cease their movements. This results in an iterative procedure of spatially orienting residents to be placed in a macroscopic set of similarly labeled clusters. In an random initial allocation on the lattice a visual depiction is expected to look like ‘noise’ when different colors are provided for each type of resident. In the final iteration when the homogeneity satisfactions are met, the grid will look ‘organized’ as groups of similarly labeled actors cluster together.

The work of [2] provides an analytical formulation of the main steps of the Schelling model and reviews the variations possible. The variations are typically introduced in order to provide different proposals for new locations of moving actors or for a modification on the determination of homogeneity satisfaction. The set of Equations provided allows for the parameterization to adapt towards the different implementations without changing significant aspects of a program. This also has a benefit in that the comparisons between different modeling approaches can be seen in a compact form. The analysis of the states provided leads to a calculation of the ‘density’ of the homogeneity of local actors across all the residents. Presented here in Section 2.1 is a concise mathematical representation of the Schelling model which is formulated in such as way as to present a convenient form to produce a set of states that can be analyzed in a manner which allows a quantification of the entropy values of these states during the simulation.

A set of random initializations of the resident labels which appear ‘organized’ would be indicative of a mistake in the implementation. The final ‘organized’ state (in that there clusters of cells sharing a spatial arrangement of the same labels) could be considered to belong to a smaller class of configurations than the ones which resemble ‘noise’, from which a simulation initially begins. The paper of [3], describes in general various class sizes of lattice state space models and provides insight into the manner for the estimations of their values of entropy. By considering the Schelling model to be a system analogous to that of an ideal gas permits a similar approach for the entropy estimation between state transitions. The approach here aims to use an estimation of sizes as a definition for the model macrostates along each time point to compute the entropy trace. The change in the entropy values for the time ordered state transitions of the Schelling model aims to provide an estimate of the uncertainty of the configurations of the spatial arrangements.

In terms of detecting segregation the cellular configurations can be observed as they change over time and be interpreted by inspection but this does require qualitative descriptions which are hard to compare between studies. Various quantitative approaches for analyzing the resulting residential configurations of the simulation trajectory exist. These approaches generally involve a specification of a functional form which captures a change in the homogeneity densities of the actor arrangements over the iterations. The application of monitoring the density of the resident satisfactions across the iterations is applied commonly in practice [2,4,5]. Given that there are *N* residents the model explores the cell occupation state changes which results in the expected satisfaction among all non-empty cells. This value can be taken according to dissatisfaction so that the value is seen to decrease over the simulation progression.

The work of [6] develops a method based upon the cluster geometry to measure the label segregation. Approaches to finding a spatial measure which captures obscure cases is an ongoing area of research and can be relied upon for specified classes of patterns [7] produces a physically inspired model for the gradient of the cluster formations so that the arrangements over the iterations resemble that in other physical phenomena but these states are still assess qualitatively by inspection as noted in [8]. From the methodology of [9] a representation for certain ‘patterns’ (specific macroscopic configurations) are considered for their size examining a range of predefined scenarios. The approach also employs a Markov chain with Metropolis transition probabilities for the change of states between and although very interesting it does deviate from the archetypical Schelling model paradigm.

In [10,11] the density function of energy differences ΔE and average magnetization 〈M〉 for different time steps of the model is shown when the Schelling model is approached as a thermodynamic system. These models provide a great deal of insight into the phase changes of the system which delivers a potentially invaluable method to detect paradigm changes in the macrostates. An accurate application to an actual sociological scenario could potentially divert large scale negative consequences. Their main strength is in determining the changes in the value of the parameters of the model which can affect the spatial arrangements under the premise that those values can be mapped to values observed in a real world scenario (a task which is another challenge in itself). To monitor the state of the system [10], uses the energy per site, ${}^{E}{/}_{N}$. The results show a reduction in the energy over the system as the residents in satisfied states will cease to move.

There are also measures to examine the motifs of the spatial arrangements for clustering patterns that are then aggregated to assess the degree that those features of interest have manifested themselves. An example of this approach is in [12] that applies the community triads (3-cycle) analysis which is supported in social science [13]. This could be considered a mesoscopic feature of the state space over the homogeneity assignments and for situations where these features are specifically important, provides a specific insight.

These differences in the measures applied to monitor the Schelling model trajectory can be seen in a comparison of the figures produced in [2,10], which represent the degree of residential homogeneity satisfaction across the lattice for the time steps. Although they have similar starting and final points, as defined by the dynamics, the trajectories followed are different based upon the measures used to evaluate the overall homogeneity between these points. As the application is motivated by residential movements, these iterations can correspond to many years with differences in values representing large changes in populations and residential prices. The entropy estimation on the trajectory of the simulation is fundamentally different from macroscopic measures built upon the aggregate microscopic state values, or mesoscopic configuration properties. By using a measure based upon the size of a set of states which are considered equivalent given the macrostate values, the investigation can answer questions regarding the ability for certain dymamics to search the configuration space. With respect to the Schelling model an estimation of the entropy values along the trajectory allows for an assessment of the degree in which a simple set of decision rules for the agents leads to configurations that are highly unlikely to be derived from random allocations.

The Schelling model of the residential population movements can be considered as a system analogous to a lattice gas [14]. This permits a similar manner of analysis in which there is a definition of microstate configurations which are analyzed as being part of a macrostate for a thermodynamic system, and therefore the entropy can be estimated for the macrostates. Given this framing of the Schelling model environment of the actors, the homogeneity threshold calculation as a discrete measure dependent upon the state values of its adjacency set can be developed to associate it with other physically focused models. The homogeneity aggregate in the Schelling lattice model can be used to develop an analogy to the Ising model [15,16,17], and Reference [18] provides a thorough formulation of the Schelling model in a manner where an evident extraction of a statistical mechanics treatment of the model simulation analysis is presented. In the Ising model, there are 2 states that represent spin, ‘up’/‘down’ ({+1,−1}). The vicinity of these states of spin alignments produces different aggregate forces for the system as do the homogeneous/heterogeneous neighbor associations in the Schelling model. The Ising model where on each location of the lattice (generalizable to *d* dimensions), it is occupied by an object with 2 states is:

$$H\left(\sigma \right)=-\sum_{i,j}\sigma_{i}\sigma_{j}.$$

This is the main starting point for the similarity of the comparisons of the Ising model with the Schelling model. The work of [8,19,20] provide examples of methodological investigations of the Schelling model with approaches derived for the Ising model. A definition for a macrostate of the Schelling model allows for a set of configurations to be considered as microstates whose estimated set size provides for a means to calculate the entropy along the iterations of the simulation.

A different operator than multiplication is in use for the lattice adjacency set to assess the equality of state label assignments. It does not follow from the same motivation of the group equality test as is described in the sociological research [21]. Equation (1) provides a functionally equivalent formulation for the 2 dimensional lattice utilizing the Kronecker delta (testing for label equality). This can easily be generalized to larger group sets without a ‘spin’ property being closely adhered to (similarly to the general feature set homogeneity summation employed in [22]). As well, the approach taken here gives an explicit representation for the empty cells which is required in the Schelling model and not the Ising model. This extra state label is excluded from contributing in the macrostate assignment as a resident lacking a threshold of satisfaction for homogeneity. A physics model interpretation is the ‘3-state voter-type non-equilibrium model on a lattice’ as described in [23].

The *equal a priori probability postulate* [24], does not apply to systems operating with these dynamics as the ergodic hypothesis does not necessarily always hold for the Schelling model. The configuration trajectory allows for the systems to become fixed without movement in the case where all residents have a sufficient number of adjacent homogeneous members in their locality. Another case is when there is a very limited number of empty cells and that explorations fail to produce an effective move. Even with increasing the number of iterations this will not change the state to produce new configurations (microstates) during a simulation. A lack of changes in the agent satisfactions from the initialization step are not conditioned for in the standard Schelling model although their addition does not impose any contradictions upon the framework. Such a situation can be treated with a terminating condition based upon the number of iterations which have not caused a change in the agent satisfactions. Another permitted case, which is ergodic, is when a limited number of residents, less than the remain threshold value for each group exist amongst a large number of empty cells so that they will be guaranteed movement in each iteration. Therefore the residents would continue to explore every microstate configuration over enough iterations (random walk over the whole state space) while failing to produce homogeneity satisfaction.

## 2. Methodology

The two subsections here outline the Schelling model implementation/variant applied in this work and the framework for the analysis of the Schelling model evolution as an entropy trace is described.

### 2.1. Schelling Model Outline

We consider a lattice Λ, of 2 dimensions (d=2) to place the hypothetical residents within cells of the lattice. The number of cells which can be occupied for a square lattice is N=|Λ| which is equal to the side length raised to the value of *d*. The lattice cells can be indexed as n∈[1,2,…,N] without taking into account the prefix ordering. According to the application of this work, each cell is occupied by a ‘resident’ which is a member of a particular group. Each cell is allocated the state of one of 3 different categories, *group1*, *group2*, and *empty*. The membership for each cell, belongs to a unique element in the set $m_{n}\in \left\{m_{group1},m_{group2},m_{empty}\right\}\forall n$ and metrics of the model can condition upon these labels as needed. The use of the label for empty units of cells, $m_{n}\in \left\{m_{empty}\right\}$, allows for there to be movement of a single resident independently of the other residents in $\left\{m_{group1},m_{group2}\right\}$. These are the only members that can affect the residency satisfaction thresholds, and whether other residents remain in the same position in the next iteration of the simulation. Simulation require that $\{m_{empty}\}\neq \emptyset$.

The mapping of a resident’s position in the lattice is denoted as $m_{n}↔m_{\left(ni,nj\right)}$ when describing the position and placement amongst cell occupants in the space of *d*. The iterations of the simulation take place over a hypothetical unit of discrete time, *t*. Since the dynamics of the system do not parameterize upon the amount of time passed, the unit of time is a representation of the steps the residents have taken since the initialization. The cell occupation of a resident can include the time index to denote the temporal locations, $m_{n,t}↔m_{(ni,nj),t}$.

The basic premise of the Schelling simulation is that each cell resident performs a check for equality of group membership upon the adjacent cells. This affects the choice for a resident to remain in the same cell between iterations. While excluding for a check against the resident’s self, it evaluates the membership of all the cells in the adjacency of a single unit. The aggregate of the equality checks can be found for $m_{n}$ via:

(1) $$l\left(m_{n}\right)=\sum_{i=-1}^{1}\sum_{j=-1}^{1}\left(\delta_{m_{\left(ni,nj\right)},m_{\left(ni+i,nj+j\right)}}:i,j\neq 0\right).$$

Here $\delta_{m_{n},m_{n}^{\prime }}$, is the Kronecker delta so that group membership equality is 1 and 0 otherwise. The examination of cell values outside of the lattice bounds are treated as $m_{empty}$ making no contribution to the residential satisfaction as this function is not called on behalf of empty cells. This state determination for each cell based upon the adjacency of the grid is the main motivation for the comparison to the Ising model in the previous research [25,26].

The aggregate of the observed equality checks is then used to determine if a homogeneity threshold is satisfied. This value, *h*, is a homogeneity constraint upon the locality of the adjacent cells that determines whether the resident in a cell will remain in the same cell or attempt a move to another one in the subsequent iteration of the simulation. The threshold for whether the resident remains in the same cell is based upon the value of local homogeneity from Equation (1):

(2) $$r\left(m_{n}\right)=\left\{\begin{array}{cc} \left(l\left(m_{n}\right)\geq h\right) & \;\mathrm{if}\;m_{n}\notin \left\{m_{empty}\right\}\\ 0 & \;\mathrm{if}\;m_{n}\in \left\{m_{empty}\right\} \end{array}\right..$$

The value for threshold is set to h=6 in this work (as in [25]), and it is typical for this to be adjusted to examine the sensitivity with the final $\left\{m_{group1},m_{group2}\right\}$ (non-empty residents) spatial arrangements. This binary value represents the state for each of the non-empty occupants for which the movement is conditioned upon. The empty set of residents are considered to not have a value of residential choice as their ‘displacement’ between time units depends upon other group states.

If $r(m_{n})=1$, the resident remains in the same cell location between time steps, $m_{n,t},m_{n,t+1}\to m_{\left(ni,nj\right)}$. In the situations where $r(m_{n})=0$, the occupant no longer remains in the same cell, there is a move to another cell location which is occupied by a resident who is a member of the ‘empty’ label, $m_{empty}$. The consecutive lattice position is uniformly chosen from those which are occupied at *t* by an $m_{empty}$,

(3) $$m_{(ni^{\prime },nj^{\prime }),t+1}\leftarrow c\left(m_{n,t}\right)=\left\{\begin{array}{cc} U\left(m_{(ni^{\prime },nj^{\prime }),t+1}\in \left\{m_{empty,t+1}\right\};r\left(m_{(ni^{\prime },nj^{\prime }),t+1}\right)=1\right) & \;\mathrm{if}\;r\left(m_{n,t}\right)=0\\ m_{(ni,nj),t} & \;\mathrm{if}\;\;r\left(m_{n,t}\right)=1. \end{array}\right.$$

This condition for the sampling requires that the proposed position replacement results in Equation (2) satisfying the threshold. The update of the set of empty labeled positions for the following time step is associated with this allocation by adding this resident’s $m_{n}$ position to the empty occupation in t+1 and removing the current position;

(4) $$\left\{m_{empty,t+1}\right\}=\left\{\left(\left\{m_{empty,t+1}\right\}\cup \left\{m_{(ni,nj),t}\right\}\right)\backslash \left(m_{(ni,nj),t+1}\right);\forall n\left(m_{(ni,nj),t}\neq m_{(ni,nj),t+1}\right)\right\}.$$

These updates to the locations of those non-empty residents with $r(m_{n})=0$, and not those of $r(m_{n})=1$ produces an iterative search for local homogenization of the lattice state space.

A simulation of the Schelling model is a trajectory of the position reassignments for the residents over the time units, which is defined by Equations (1)–(4). The updates are performed by the assignments in Equations (3) and (4). For the complete sequence per time step, a cycle through each node is required. Let $o_{t}$ be a random variable following the discrete uniform distribution over the set [1,2,…,N] sampled without replacement for each time point. The update is:

(5) $$m_{o_{n,t},t+1}\leftarrow c\left(m_{o_{n,t},t}:\forall n\in \left[1,\ldots ,N\right]\;m_{o_{n,t},t}\notin \left\{m_{empty}\right\})\right),$$

and the remain state for residents over the course of the simulation can subsequently be given by:

(6) $$r_{t+1}=\left[r\left(m_{1,t+1}\right),\ldots ,r\left(m_{n,t+1}\right),\ldots ,r\left(m_{N,t+1}\right)\right].$$

This gives an expression for the state of every resident in the lattice and the coordinates that are occupied for the indexed time unit. Given that the allocation of a position for which a resident can change their state of ‘remain’, from 0 to 1, arises from this sampling procedure; this results in different trajectories of the simulation upon multiple realizations.

### 2.2. Estimating the Entropy of the Schelling Model from the Microstate and Macrostate Assignments

Using the formulation of the Schelling model in Equations (1)–(6) a representation of the model state space as a closed thermodynamic system can be provided. The simulation is modeled as a microcanonical ensemble where the microstates are the configurations of the binary valued entries of each resident’s ‘remain’ value for the set of all lattice members in $r_{t}$ (Equation (6)).

The macrostate, denoted by *R*, of the Schelling model will be defined as the aggregate remain value over all the resident members:

(7) $$R=\sum_{n=1}^{N}r\left(m_{n}\right).$$

This can be indexed to derive the macrostate value for each time point as $R_{t}=\sum_{n=1}^{N}r\left(m_{n,t}\right)$. For the different states of remain that the residents produce this macrostate effectively groups the microstates by the local homogeneity satisfaction over all non-empty members. This aggregate can be satisfied by multiple configurations and the size of the sets which satisfy different values of *R* will be estimated via a sampling procedure. *R* represents an intuitive macrostate property that can be interpreted and the number of corresponding microstates are of interest for modelers to examine the extent to which the system follows various microstate explorations. The Schelling model phase space is therefore described here as having this 1 degree of freedom.

The number of microstate configurations which corresponds to a particular macrostate, *R*, is represented as, $\Omega \left(R\right)$. It is assumed that each microstate $r_{t}\in R$ has an equal probability of being selected in the macrostate; $r_{t}{=}^{1}{/}_{\Omega \left(R\right)}$. The entropy of the macrostate can then be found via:

(8) $$S=k_{B}\ln\Omega \left(R\right).$$

The value of Ω is found by enumerating all of the microstates which produces the same remain level of *R* for the set of residents. For a particular time point, the entropy for the macrostate of the Schelling model can be addressed as $S_{t}=k_{B}\ln\Omega \left(R_{t}\right)$.

Given that the enumeration for the microstate space of the ensemble is not analytically tractable, with the state positions depending upon discrete non-linear operations, $\Omega \left(R\right)$ will be sampled with Monte Carlo [27] (similar manner employed in [18,23], and thoroughly described in [28]). The Monte Carlo sampling scheme draws from the space of Equation (6), $U\left(r\right)$. The size of the collection of microstates for all macrostates, is equal to:

(9) $$\sum_{R=R_{min}}^{R_{max}}\Omega \left(R\right)=\frac{N!}{\Pi_{g=1}^{∥group∥}N_{g}!}.$$

$N!/(\Pi_{g}^{group}N_{g}!)$, is the de-labeling factor, *N* the number of positions in the lattice and $N_{g}$ the number of agents (members) within each group. The lattice structure is represented as an adjacency list so that the different microstates can be seen as a rearrangement upon an array which the de-labelling factor removes redundant permutations from as they do not change the macrostate assignment.

It is of particular interest to obtain the trace of the entropy values of the model during the time steps. These values $S_{t}$, depend upon $\Omega (R_{t})$ which will be proportions of the overall count from all macrostate sizes in Equation (9). This is because each non-redundant permutation of the labels will correspond to a particular value of *R*,

(10) $$\tilde{\Omega (R)}=P\left(R\right)\times \frac{N!}{\Pi_{g=1}^{∥group∥}N_{g}!}.$$

The Monte Carlo sample for the probability of a microstate belonging to a particular macrostate is effectively the macrostate membership assignment ratio for a sample size *k*; |{r∈R}|/k. This allows for an estimation of *S* and at every time point ${\tilde{S}}_{t}=k_{B}\tilde{\Omega (R_{t})}$. The convergence of the sample distribution over *R* (via Monte Carlo on the space of r) is tested by examining the first half of the samples against the second half and performing a K-S test. This allows for the Schelling model trajectory to have every lattice configuration mapped to a macrostate value through Equation (7) and the corresponding entropy value to be produced based upon the estimate of the number of microstates. Alongside the visual representations of the lattice configurations over time with color codings for the different actors, an entropy trace can be produced.

## 3. Results and Discussion

This section explores the Schelling model in terms of the macrostate value at each iteration defined in Equation (7) and the entropy value estimated ${\tilde{S}}_{t}=k_{B}\tilde{\Omega (R_{t})}$. In the examples used N=100, and 2 types of non-empty agents are considered $m_{agent1}$ and $m_{agent2}$ each set having 45 members. The members of the empty set are allocation subsequently as a default allocation to those cells which a label is unassigned so that $m_{empty}$ has 10 members in these cases.

The Monte Carlo sampling scheme required for Equation (10) is computationally demanding. It is possible to obtain reliable convergence on the estimated macrostate densities given new processor speeds, and the parallel processing capabilities readily available. The methodology is implemented in Julia-Lang [29,30], which is a relatively new programming language designed for high performance computing in mind. The computations were parallelized with minimal syntactic additions to the single threaded implementation via the intuitive programming macros which can encapsulate regions that require parallel operations.

**Figure 1:**
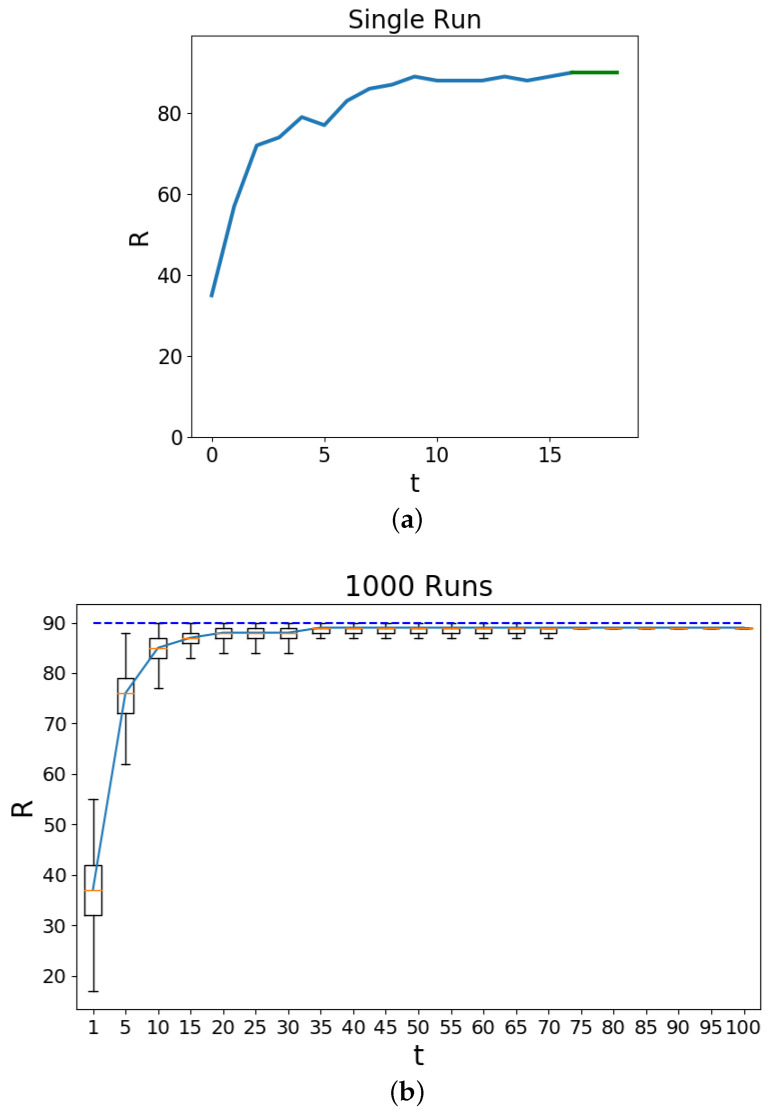
These figures examine the value of the macrostates $R_{t}$ (Equation (7)) over the course of the Schelling model simulation. The time *t* is the number of iterations and the value of *R* is the total number of agents which have their locality homogeneity requirement satisfied at h=4. Here N=100, $|m_{agent1}\left|=45,\right|m_{agent2}|=45$ and $m_{empty}=10$. Subfigure (**a**) shows an example single run of the simulation macrostate, $R_{t}$, values over time. The green line denotes the region where there are no more changes to the *R* values. Subfigure (**b**) shows the macrostate, $R_{t}$, values over a set of 1000 runs with box plots (5-point statistics). The dashed line shows the theoretical maximum value that could be achieved.

Figure 1 examines the value of the macrostate *R* at every time point (iteration) in the Schelling model. In Subfigure (a) a single run of a simulation is presented where it can be seen that the aggregate value of *R* increasing until stabilization at the value drawn in ‘green’. This green line component represents the time points where no movement of the agents can change their state of $l(m_{n})$ (Equation (1)). The occasional increases and decreases emphasizes the aspect of the simulation dynamics of how the random uniform allocation of the new positions does not guarantee improvements upon the macroscopic state where the threshold satisfactions over the complete lattice are to be monotonically increasing. Subfigure (b) presents the results from 1000 independent simulations of the model with box plots for the values encountered at every iteration. Towards the end of the simulation the independent simulations maintain the same values. The dashed line represents the maximum value that could be achieved which is the total number of non-empty residents (90). It can be seen that as the simulation progresses the macrostate value of *R* also increases with the residents changing positions until a cell with *h* or more similarly labeled residents surround it. As *R* is the aggregate of these satisfactions across the lattice it can be expected that the dynamics provide an avenue to increase this macroscopic measure. In (b) the spread between the maximum *R* values for the first iteration (initialization) does not surpass the median value of the 5th iteration. This highlights the effectiveness of the dynamics to search for large *R* values in contrast to a random sample and that large *R* contain fewer microstates r.

**Figure 2:**
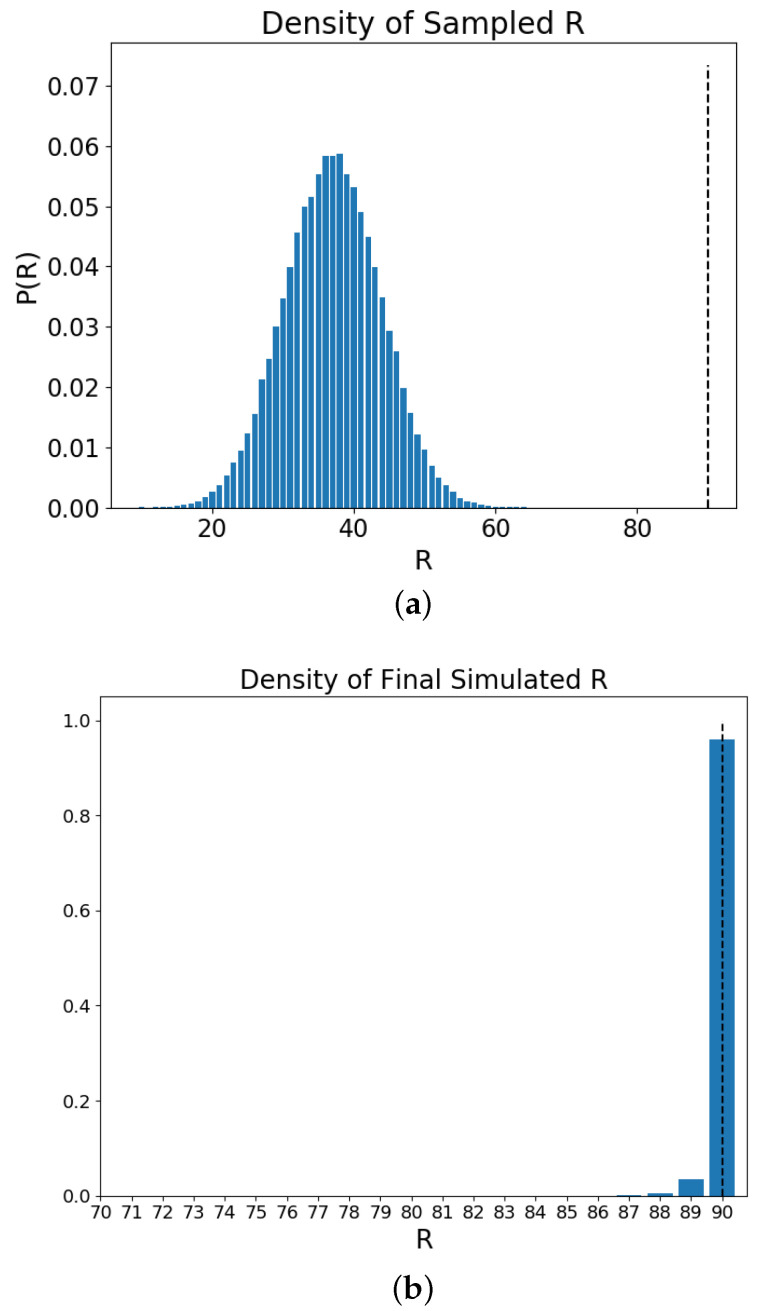
The Schelling model used here has a lattice size of N=100 and non-empty agents of $|m_{agent1}|=45$ and $|m_{agent2}|=45$ with the empty being $m_{empty}=10$. The simulations were run 10K times with independent initializations. Subfigure (**a**) shows the results from a Monte Carlo sampling scheme for the values of the macrostate value *R* (Equation (7)), based upon independently drawn initial configurations of the residential actors of the Schelling model. The density of the different *R* values can be seen in the height of the bars proportional to the observations of that value. The dashed line represents the largest value of *R* obtainable for this simulation setup; 90. Subfigure (**b**) shows the final values of *R* for a set of independent Schelling model simulations which have been allowed the necessary time steps until their configurations remain static. The dashed line represents the maximum possible *R* value in this setup. (Both simulations were run with 10K runs and tested for convergence)

Figure 2 shows the results of examining the macrostate values *R* (Equation (7)) from independent simulations of the Schelling model at the start (initialization) and at the end (final value). Subfigure (a) shows the Monte Carlo sample distribution of the *R* values and also displays the maximum possible value R=90 in the dashed vertical line. Each possible value that can be sampled is given an initial observation count of 1 so that comparisons with states the simulation enters that are rare to sample can still be compared. Each initialization is independent of the previous ones and aims to depict the relative size of the different macrostate values. It can be seen that sampling from the microstates the values close to the maximum can be expected to require a large number of draws. In terms of the Schelling model, and the interpretation of the *R* value, it can be said that there are more random initializations (microstates) which have less than half of the agent thresholds satisfied. Subfigure (b) presents the final *R* value at the end of a set of independent simulations. The final value is determined by a stretch of iterations in which the *R* value does not change or that the maximum possible value has been reached. Although the microstates which correspond to such large values of *R* are encountered in a small fraction of the Monte Carlo simulation, they are arrived at consistently, given the iterations of the simulation dynamics. The Schelling model is therefore effective in navigating the space to find microstates which correspond to large *R* values.

**Figure 3:**
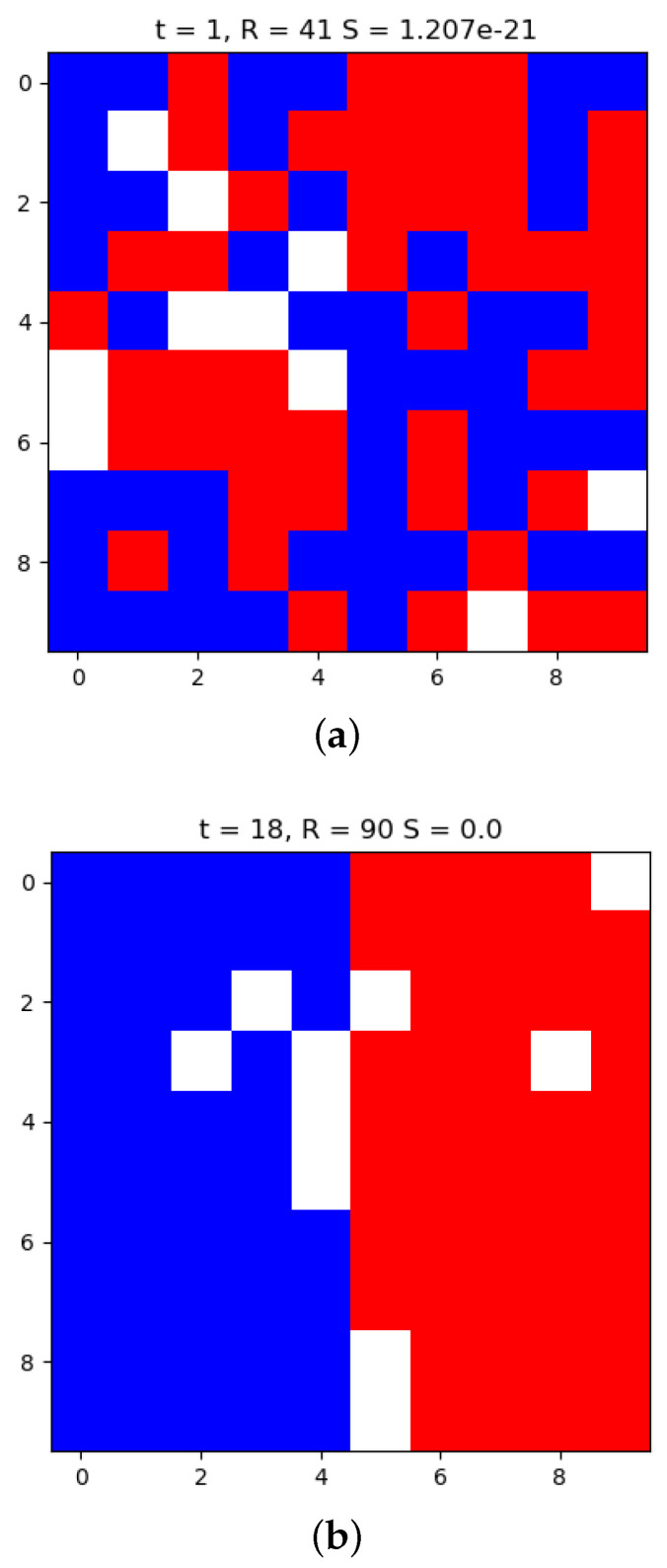
The Schelling model is simulated for a lattice size of N=100, non-empty agents of 45 members each and 10 empty cells. Over the course of a simulation the lattice view is displayed at the initialization, t=1 in Subfigure (**a**), and the time point where the repositioning of the residents ceases, t=18 in Subfigure (**b**). The titles report the time point that the lattice depicted was taken from, the macrostate value *R* (aggregate number of homogeneity satisfied residents), and the entropy value $S_{t}$ (Equation (8)). The Schelling model effectively manages to reduce the entropy of the macrostate *R* to zero by the end of the simulation.

Figure 3 displays the placement of the residents in the lattice at 2 different time points in the simulation of the Schelling model. Subfigure (a) shows the state at the initialization t=1 where R=41 and the entropy calculated using Equation (8) is $S_{t}$ = 1.207 × 10${}^{-21}$. Subfigure (b) shows the resulting state of the lattice after the *R* value no longer changes and in this case the maximum possible value has been reached for this model setup (R=90). At t=18 the entropy is found to be $S_{t=18}=0$ which denotes that the number of microstates for this macrostate is 1. With no uncertainty about the microstate given the macrostate this number is understandable given that a number of 1 observation was provided for each possible observation in the Monte Carlo simulation. Since there were no microstates drawn for R=90, the density remained at 1 which produces zero entropy.

**Figure 4:**
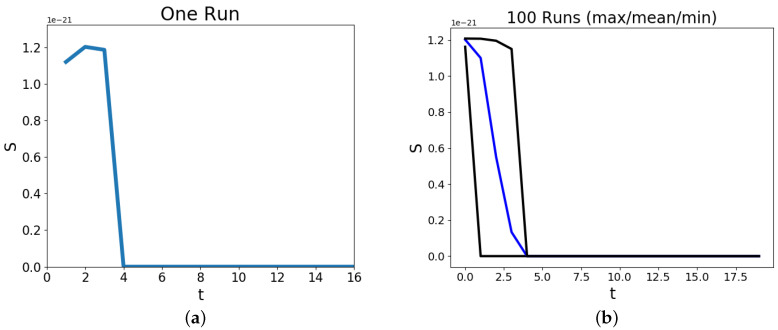
The plots show the trace of the estimated entropy values across time ($S_{t}$ Equation (8)) for random initializations of the Schelling model of a 10 × 10 lattice. There are 2 groups of residential occupants of cells with 45 members each and the remaining 10 are occupied by a group categorized as ‘empty’. Subfigure (**a**) shows the entropy trace of a single randomly initialized Schelling model. The simulation has reached a configuration of the residents where there is no subsequent movement, after t=4. The value of the entropy drops to zero and remains at that value. Subfigure (**b**) shows the maximum (black), mean (blue) and minimum (black) values of the entropy over 100 independent simulations. It can be seen how there is a spread over the entropy values for each time point although the model simulation successfully finds the minimum entropy value.

Figure 4 presents the entropy values estimated for the time steps in the Schelling model simulations. Each run is began as an independent initialization of the grid for 2 groups of residential members with 45 residents and 10 empty in a 10 × 10 lattice. These figures provide insight into the entropy values the simulation produces by state changes between iterations. The entropy values, $S_{t}$, are found from Equation (8) and are computed for each simulation independently. Subfigure (a) displays the trace of $S_{t}$ values for a single simulation. The rise and then decrease shows that the random allocation of residents by uniformly allocating new positions for those with less than the threshold of necessary homogeneity does not guarantee a macroscopic aggregate state of entropy or *R* (as displayed in a) of Figure 1. The zero entropy state is what the simulation arrives to and remains at, as there is only a single microstate member counted for that value of *R*. From the progression of *R* in Figure 1 and the distribution of the sampled values of *R* in Figure 2, it can be seen that the larger *R* values the Schelling dynamics produces are of a smaller ensemble which results in the decrease in entropy. Subfigure (b) shows the results from 100 independent simulations where the maximum, mean and minimum values of the entropy $S_{t}$ (Equation (8)) are drawn. All the simulations reach the zero entropy and it can be seen how some simulations arrive at that value requiring less or more time points. This can be due to the initializations and the sequence of random allocation of moving residents.

## 4. Discussion

The work presented here provides a formulation of the Schelling model of social segregation [1] and a manner in which the states of the simulation can be analyzed with the framework of statistical mechanics. From the concise set of equations for the dynamics of the Schelling model iterations it is seen that there is a resemblance with that of the Ising model [15] which is also noted in previous research [15,16,17,18,25]. This similarity rests predominantly upon the premise that both models utilize a lattice for the placement of cell occupants whose state depends exclusively upon the states of the occupants in the adjacent/neighboring cells. The states of the cells are updated based upon the satisfaction of the aggregate of similarity (homogeneity) counts for the ‘values’ of the occupants and the surrounding cells. In the Ising model these states are defined by ‘spins’ given binary values that are aggregated in order to define a state transition. For the Schelling model these states are represented by societal identifiers (categorical variables) which can possibly be extended to arbitrarily sized sets and it therefore is a more direct translation of the model motivation to employ the use of the Kronecker delta as shown in Equation (1). This can be understood by the residential actors performing a label equality check whose satisfaction upon the adjacency set is compared to a threshold which determines the cell state.

Equations (1)–(5) are the Equations for the states of the lattice which governs the residential system state changes between time points (iterations). Each time point the lattice has a particular state defined by Equation (6), that produces a $r_{t}$, which is the set of the homogeneity satisfactions for all the residential members of the lattice. These configurations can be treated as ‘microstates’ which change according the dynamics of the Schelling model. A ‘macrostate’ for the state of system is defined upon the microstates in Equation (7) producing a phase space with a single degree of freedom *R*. The macrostate value is the aggregate homogeneity satisfaction within the lattice and provides insight into a valuable macroscopic aspect of the system. Equations (9)–(10) describe the approach to estimating the entropy values for the simulation along the time points based upon the macrostates *R*.

Figure 4 shows that the Schelling simulation upon randomly initialized residential placements upon the lattice acts to reduce the entropy value over time. These expected transitions into smaller ensembles, based upon the simulations, show that although there are sporadic deviations from the decrease in entropy; the dynamics of the system reliably reduce the entropy. From Figure 3 the initial time point and a latter time point provide an intuitive interpretation for this by the noticing the increase in *R* along with the organization of the 2 residential groups. To see how the simple dynamics of the model are able to navigate the space and obtain it with consistently low density high values of *R*, Figure 2 shows the distribution of *R* from the initializations and the *R* values at the end of the simulation. It is intriguing how the model dynamics, representative of residential movement patterns, produces a trajectory that goes against the arrrow of time.

Future work would entail expanding upon the model to take into account the cost/energy required to perform actions such as change position or assess the aggregate value of the homophily in a cell’s locality. The subsequent investigations could look into the limit case in 1 dimension as explored in [8], and produce phase diagrams for the transitions based upon the homogeneity threshold values. The current implementation has a cubic run time in the number of grid cells for each iteration which may be improved upon using different programming data structures.

## Appendix A. Note on the use of the Terminology for the Agent Satisfaction

It should be noted that there are alternative re-phrasings for the underpinning interpretation of the model outcomes, especially to highlight the phobias the agents can be acting out upon as an explanation for their decision to change position. As most models describe the dynamics of residents reaching stationarity as the sufficient aggregate of their locality for homophilic label assignments being discovered; this is a maximization process. This could be rewritten as minimization of a cost associated with heterogeneity label assignments where the remain decision (Equation (2)) has the comparator facing in the other direction to be based upon a heterophobic threshold on the aggregate of locality assignments from Equation (1), for disimilarity. Although this may not appear to be an error the that comparator threshold is written for a maximization and the language alludes to a minimization, it is misleading. This can be seen as justifiable in the effort to attract attention towards this area of research by choosing sensitive language. The degree to which it is an actual error is equivalent to referring to an application of Reinforcement Learning (RL), that searches to maximize a reward function, but is actually emphasizing that the state sequences are optimally reducing a cost. For this reason it is considered that there is a set of dynamics which the agents follow as a search mechanism for a satisfaction of this homophilic dependency or preference.
